# Managing Pneumothorax in Neurofibromatosis Type 1: A Report of a Rare Case

**DOI:** 10.7759/cureus.76983

**Published:** 2025-01-06

**Authors:** Mouad Gourti, Mouhsine Makloul, Elmehdi Maidi

**Affiliations:** 1 Surgery Department, Medical University of Agadir, Agadir, MAR

**Keywords:** neurofibromatosis associated diffuse lung disease, neurofibromatosis type 1 (nf-1), pneumothorax (ptx), rare genetic diseases, video-assisted thoracoscopic surgery (vats)

## Abstract

Neurofibromatosis is a rare genetic disorder with variable manifestations, primarily involving neural and connective tissues. Although pulmonary complications such as pneumothorax are uncommon, they can significantly impact patient outcomes. We report the case of a 48-year-old male with a 10-year history of neurofibromatosis who presented with acute thoracic pain and dyspnea. Imaging revealed a pneumothorax requiring urgent thoracic drainage. Subsequent surgical exploration via thoracoscopy revealed apical bullae and vascularized adhesions, along with multiple intact cystic formations in the lung parenchyma. Surgical management included apical bullectomy using a mechanical stapler and talc pleurodesis. The patient’s postoperative recovery was uneventful, with complete lung re-expansion confirmed on imaging. This case underscores the importance of tailored surgical intervention in managing rare complications of neurofibromatosis, emphasizing thoracoscopy as a minimally invasive and effective approach.

## Introduction

A genetic condition known as neurofibromatosis is typified by the emergence of both benign and malignant tumors as well as a number of systemic symptoms that impact the skin, nervous system, and occasionally internal organs. Although there is ample evidence linking it to neurocutaneous lesions, pulmonary involvement - specifically, pneumothorax - remains an extremely uncommon consequence. Although the etiology behind this manifestation is not well understood, it is believed to entail anatomical abnormalities in the lung parenchyma and connective tissue [1]. Because atypical lung pathology, such as cystic lesions or adhesions, may be present in patients with neurofibromatosis, spontaneous pneumothorax poses special diagnostic and treatment issues. To treat acute respiratory distress and avoid recurrence, these patients frequently need customized care techniques [1,2].

In our study, we analyzed the clinical, radiological, biological, evolutionary, and therapeutic characteristics of our patient while incorporating a comprehensive discussion based on a review of the literature.

## Case presentation

We report the case of a 48-year-old male treated for neurofibromatosis type 1 (NF1), diagnosed in early adulthood and with no history of tobacco use. The patient had previously undergone neurosurgical intervention 10 years prior for cerebral neurofibromatosis and experienced a spontaneous pneumothorax located in the right lung 20 years earlier, which was managed with chest tube drainage. He presented to the emergency department of our hospital with acute chest pain and dyspnea.

**Figure 1:**
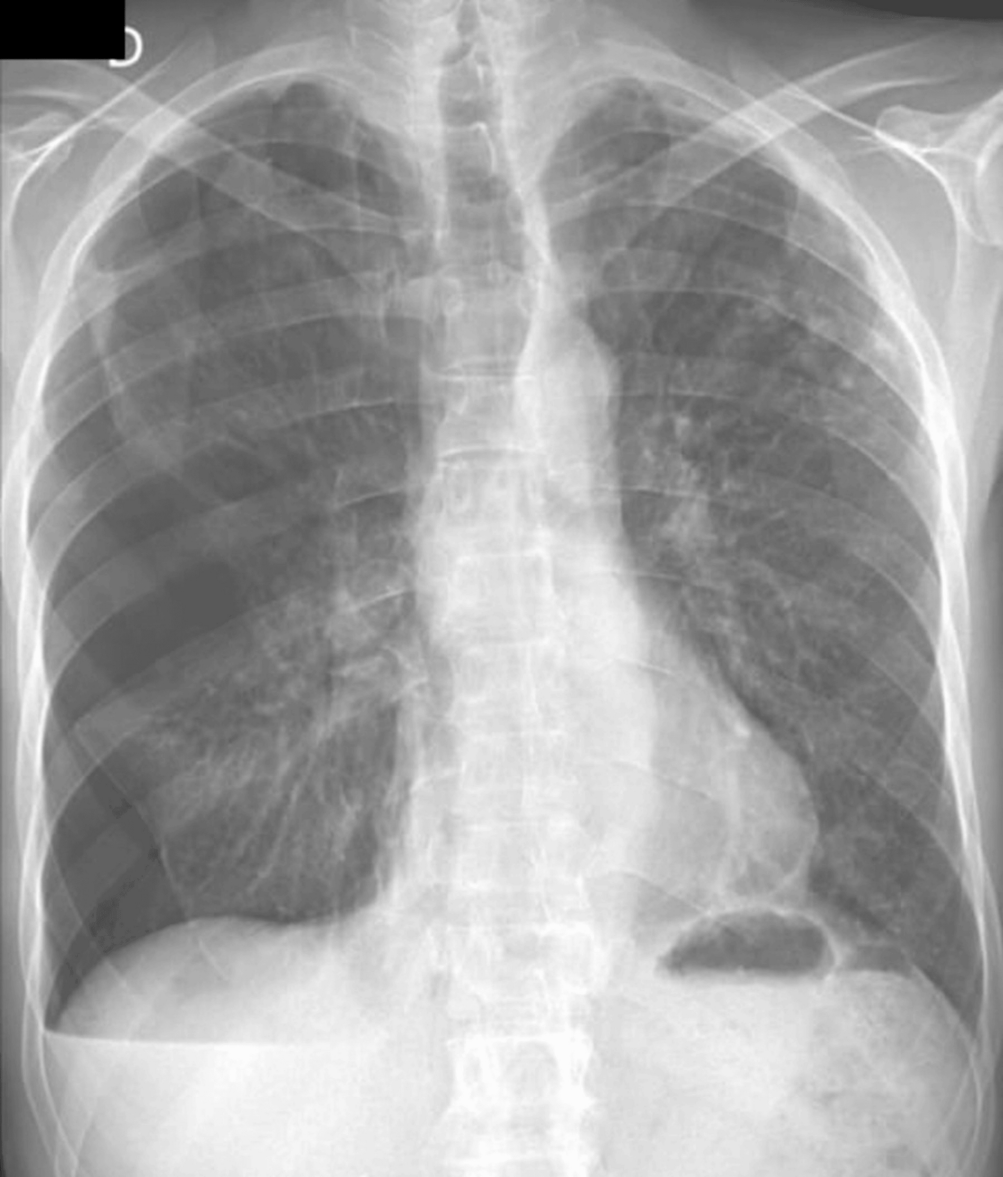
Thoracic X-ray in the postero-anterior (PA) view demonstrating basal pneumothorax

**Figure 2:**
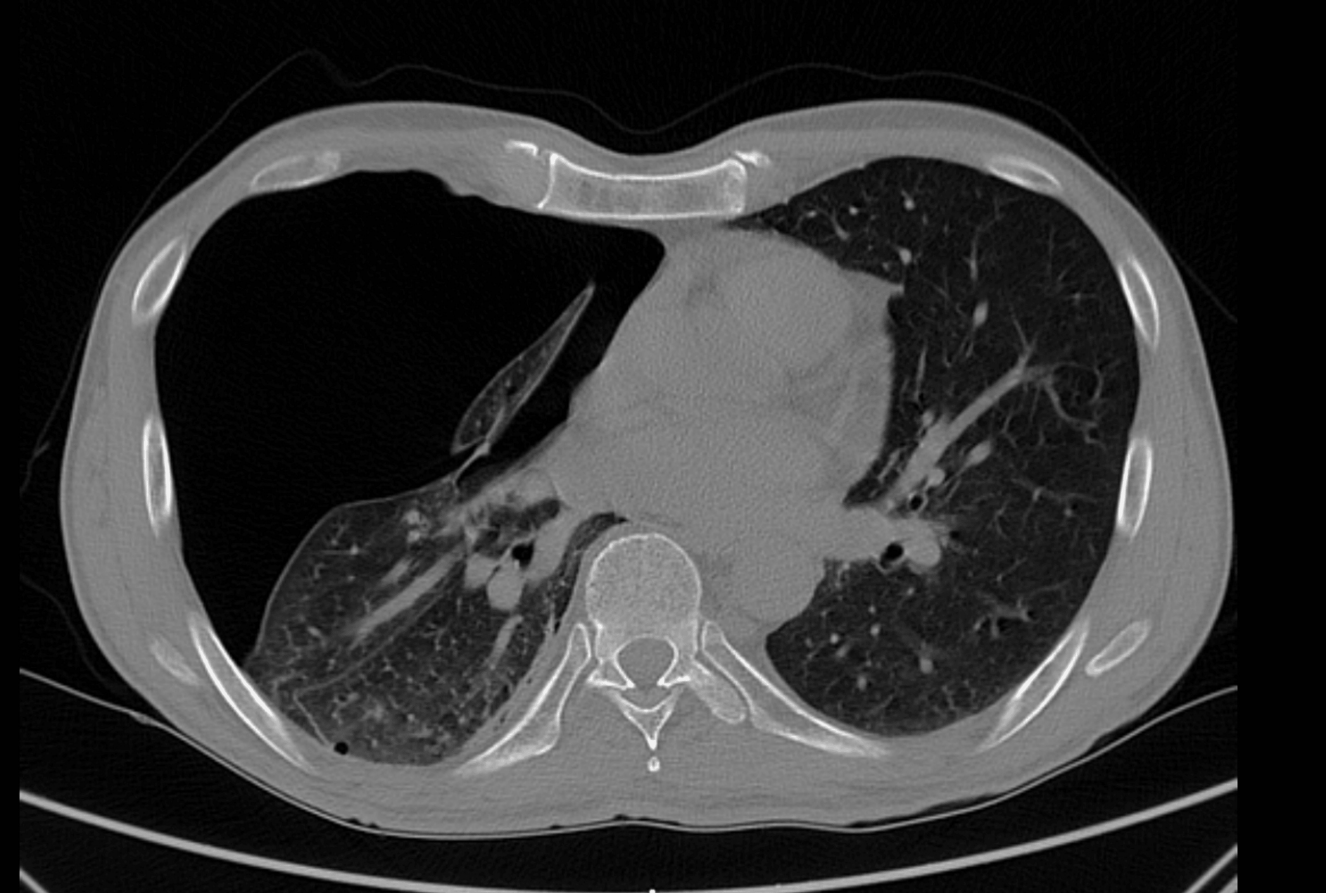
Thoracic computed tomography revealing complete pneumothorax on the right side This thoracic computed tomography showing a complete right-sided pneumothorax with partial lung collapse, without evidence of additional acute parenchymal abnormalities.

On clinical examination, the patient was hemodynamically stable but exhibited decreased breath sounds on the right side. Imaging studies, including a chest radiograph and computed tomography (CT) scan, revealed a complete right-sided pneumothorax with partial lung collapse (Figures 1-2). No additional acute parenchymal abnormalities were identified. Initial management consisted of chest tube insertion via an axillary approach under local anesthesia. A 20 Fr chest tube was placed, and negative suction at -8 mmHg was applied, leading to a good re-expansion of the pulmonary parenchyma.

Considering the patient’s history of neurofibromatosis and the recurrent episodes of pneumothorax, a multidisciplinary discussion was held involving teams from pulmonology, anesthesiology, and thoracic surgery. It was collectively decided to proceed with surgical intervention to address the underlying pathology and minimize the risk of recurrence. The patient, who had a normal respiratory function test, was managed with chest drainage for six days and underwent surgery on the seventh day.

**Figure 3:**
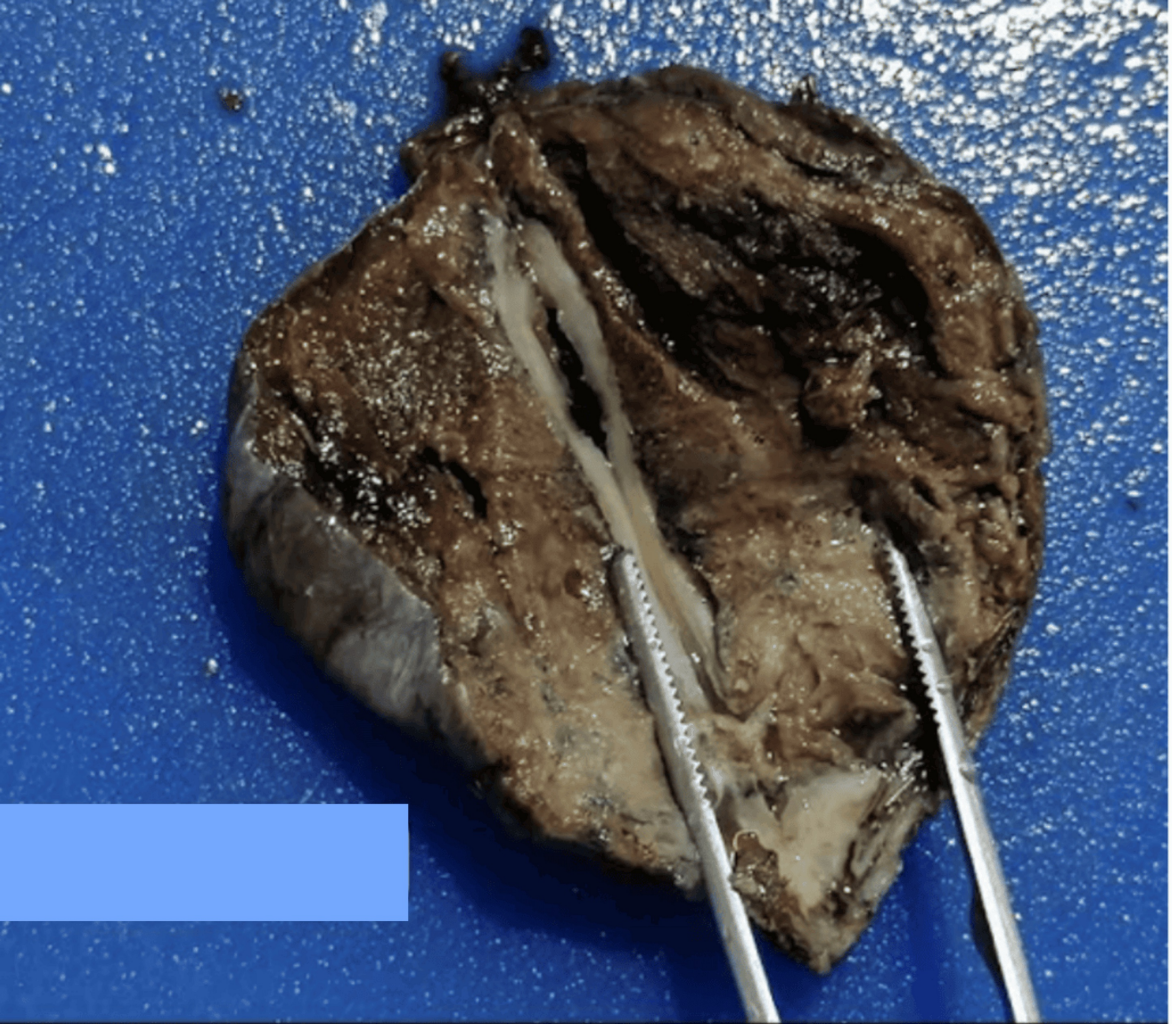
View of the resected lung parenchyma of the patient This image depicts the resected apical parenchymal tissue containing ruptured cystic bullae. These pathological changes were identified as the cause of the patient's pneumothorax.

The patient underwent video-assisted thoracoscopic surgery (VATS) under general anesthesia with selective intubation. To optimize postoperative pain control, intercostal and peri-cicatricial infiltration with bupivacaine was administered. He was positioned in the left lateral decubitus position. A two-port thoracoscopy was performed; intraoperatively, a completely collapsed lung was observed, with multiple highly vascularized adhesions and a lung filled with numerous cystic formations, as shown in the attached images. The apical largest air cyst was resected, and pleurodesis was performed using talc insufflation (Figure 3). The procedure concluded with the placement of a 28 Fr chest tube for postoperative drainage. Local anesthetic (bupivacaine) was injected at the trocar sites to manage postoperative pain. The patient’s recovery in the operating room was uneventful, with immediate extubation and normal transfer to the recovery room as per the department protocol. Subsequently, the patient was transferred to the surgical hospitalization unit.

**Figure 4:**
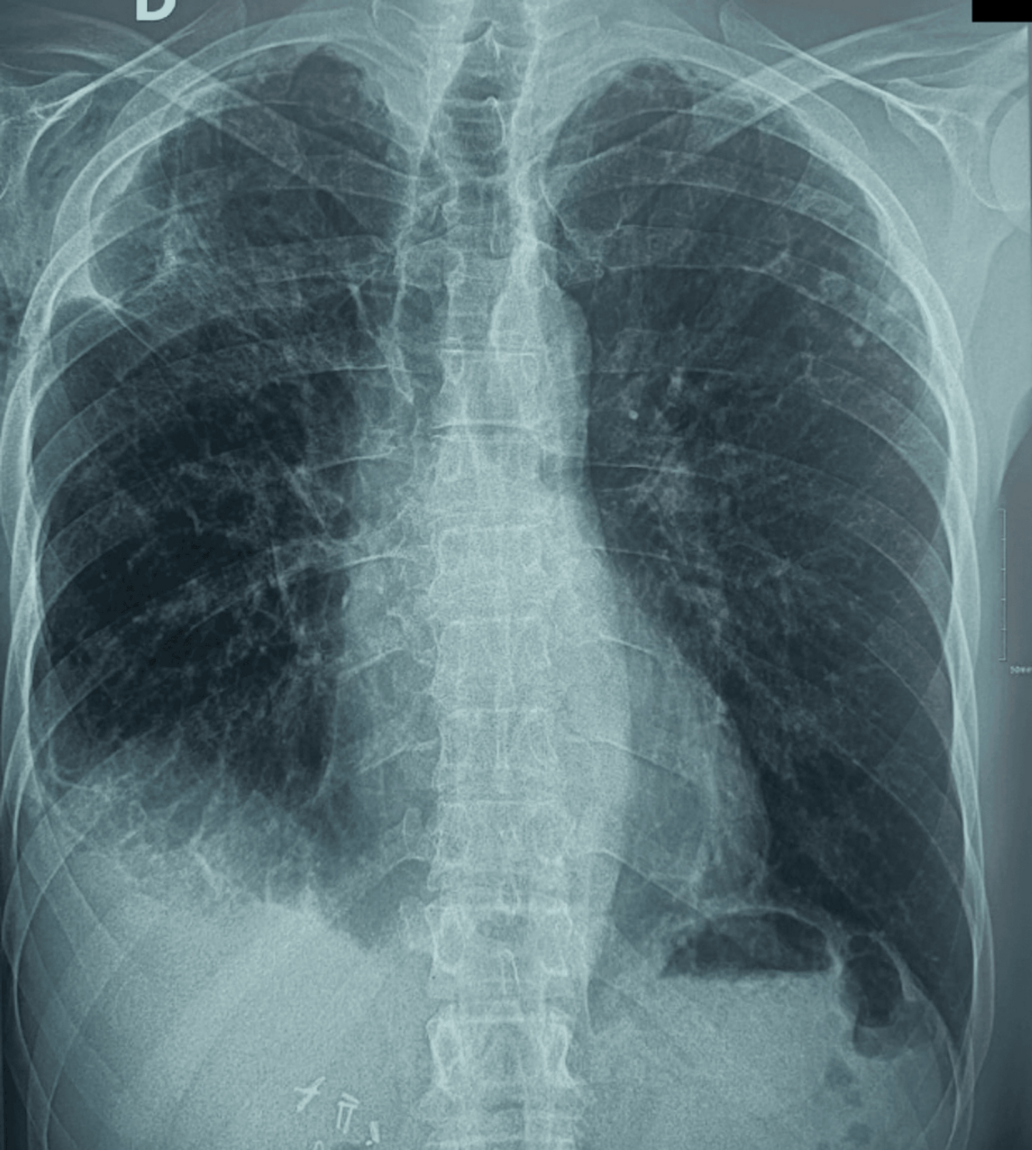
Postoperative chest X-ray of our patient Postoperative chest X-ray showing normal findings in our patient after surgery.

Postoperatively, the patient’s recovery was uneventful. The chest tube was removed on postoperative day 3 following complete lung re-expansion and cessation of bubbling. The patient was discharged on postoperative day 5 in stable condition with no residual symptoms. Follow-up imaging at one month confirmed full resolution of the pneumothorax and no recurrence (Figure 4).

## Discussion

NF1, also known as von Recklinghausen's disease, is an autosomal dominant disorder with a prevalence of 1 in 3,000 individuals [1]. While it primarily affects the nervous system, it also involves ectodermal and mesodermal tissues, with variable clinical manifestations. Thoracic and pulmonary complications, though less commonly recognized, can significantly impact morbidity [2]. These include cutaneous and subcutaneous neurofibromas on the chest wall, thoracic neoplasms, and interstitial lung disease (ILD).

The diagnosis of NF1 is established based on the presence of two or more of the following clinical criteria [1,3]: six or more café au lait macules, two or more neurofibromas, or one or more plexiform neurofibromas; skinfold freckling in the axilla or groin, optic pathway glioma, two or more Lisch nodules, distinctive bony dysplasia, such as sphenoid wing dysplasia or thinning of the long bone cortex. A first-degree relative with NF1. These criteria reflect the diverse manifestations of the disease, ensuring accurate and early diagnosis (Table 1) [1,4].

**Table 1:** Diagnostic criteria for neurofibromatosis type 1 (NF1) NF1: neurofibromatosis type 1 Reference: [1]

Diagnosis of NF1 can be made in an individual with two or more of the following criteria:
≥6 café au lait macules (> 5 mm in greatest dimension for prepubertal persons and > 15 mm in greatest dimension for postpubertal persons)
Axillary or inguinal freckles
≥2 neurofibromas (any type) or 1 plexiform neurofibroma
Optic glioma
≥2 Lisch nodules
Sphenoid dysplasia, tibial pseudoarthrosis, or other distinctive bone lesion
First-degree relative with a diagnosis of NF1

Diffuse lung disease in NF1, though rare, has been documented in the literature. It is generally bilateral and symmetrical and predominantly affects the basal regions of the lungs. It is often characterized by thin-walled bullae located in the upper lung zones, accompanied by lower lobe fibrosis. This combination has been considered a hallmark of NF1-associated lung disease, although it is not pathognomonic of the condition [5,6]. The exact prevalence of this association remains unclear, and its relationship with risk factors such as smoking is still debated [3,5,7].

The lung is indeed a potential site of involvement in neurofibromatosis. This entity, first identified in 1963 [5,8], has been characterized in numerous studies. This association typically presents with dyspnea and shortness of breath on exertion, with pulmonary function tests revealing obstructive or restrictive defects, along with consistently decreased diffusion capacity of carbon (DLCO) [4]. Radiographic imaging often shows apical asymmetric thin-walled bullae and subpleural reticular abnormalities, though more advanced patterns such as honeycombing, akin to idiopathic pulmonary fibrosis, remain exceptionally rare [5,8]. In our patient, the thoracic CT scan revealed a complete right-sided pneumothorax with partial lung collapse, but there was no evidence of additional acute parenchymal abnormalities. These findings highlight the variability of pulmonary manifestations in NF1.

In this context, the description of pneumothorax associated with neurofibromatosis is an even rarer occurrence. While the development of bullae in neurofibromatosis predisposes patients to pneumothorax, its clinical presentation and management in such cases remain poorly documented. Additionally, the surgical approach to pneumothorax in patients with neurofibromatosis lacks detailed exploration in existing literature [5]. This underlines a significant gap in understanding the optimal management strategy for this rare and potentially severe complication of neurofibromatosis.

In cases of recurrent pneumothorax associated with neurofibromatosis, it is crucial to consider differential diagnoses, particularly genetic disorders that share overlapping clinical features. One such condition is Birt-Hogg-Dubé syndrome (BHDS), a rare genetic disorder characterized by fibrofolliculomas, renal tumors, and lung cysts, which predispose to spontaneous recurrent pneumothorax. While BHDS and neurofibromatosis can both present with recurrent pneumothorax, genetic testing and clinical evaluation play a pivotal role in distinguishing between these conditions [1,7]. These findings underscore the importance of exploring genetic testing and a comprehensive evaluation of all potential diagnoses in patients with recurrent pneumothorax, as the underlying condition significantly influences management strategies and long-term outcomes.

In our case, the patient presented with recurrent pneumothorax, an uncommon but significant pulmonary manifestation of NF1. Intraoperative findings of multiple pulmonary cysts, extensive adhesions, and emphysematous changes are consistent with previously described thoracic complications of NF1 [1,5]. These structural abnormalities likely predisposed the patient to recurrent pneumothorax. The presence of dense vascularized adhesions further suggests a chronic inflammatory response, potentially exacerbated by previous episodes of pneumothorax and underlying pulmonary fragility.

The management of NF1-related complications remains a rapidly evolving field, with emerging therapies offering potential improvements in outcomes. Recent studies have highlighted promising avenues for future exploration; early-phase data have demonstrated the efficacy of selumetinib, an MEK inhibitor, in reducing the size of inoperable plexiform neurofibromas in children with NF1. This long-term treatment showed significant benefits in tumor control and quality of life, paving the way for its application in other NF1-associated complications [9]. Preclinical studies suggest that malignant peripheral nerve sheath tumors (MPNSTs) in NF1 may respond well to combined therapy using all-trans retinoic acid (ATRA) and MEK inhibitors. This dual approach has been shown to enhance therapeutic outcomes and offers a promising direction for addressing this aggressive tumor type [10]. Preconception genetic counseling remains a cornerstone in the care of adult NF1 patients. This strategy, recommended for all individuals with NF1, provides crucial insights into disease transmission and assists patients in making informed reproductive decisions [11].

This case underscores the need for a multidisciplinary approach to managing complex thoracic manifestations of NF1. Collaboration between pulmonologists, thoracic surgeons, and anesthesiologists ensures optimal outcomes. Additionally, further studies are necessary to clarify the pathophysiological mechanisms and to establish guidelines for the diagnosis and management of NF1-related pulmonary complications.

## Conclusions

The lungs can be affected in NF1, though manifestations such as pneumothorax are rare. The absence of well-defined surgical guidelines for managing pneumothorax in this context underscores a significant gap in the literature. Through this documentation, we aim to enhance our understanding of the clinical challenges and management strategies associated with neurofibromatosis-related pulmonary complications. Future research is essential to establish standardized diagnostic and therapeutic pathways, ultimately improving outcomes for these complex and uncommon cases.
